# Evolution and multiple origins of zona pellucida genes in vertebrates

**DOI:** 10.1242/bio.036137

**Published:** 2018-11-15

**Authors:** Jin-Mei Feng, Hai-Feng Tian, Qiao-Mu Hu, Yan Meng, Han-Bing Xiao

**Affiliations:** 1 Department of Pathogenic Biology, School of Medicine, Jianghan University, Wuhan, Hubei Province 430056, China; 2 Department of Aquaculture and Genetics Breeding, Yangtze River Fisheries Research Institute, Chinese Academy of Fishery Sciences, Wuhan 430223, Hubei Province, China

**Keywords:** Zona pellucida, Egg coat, Vertebrate, Evolution

## Abstract

Animal egg coats are composed of different glycoproteins collectively named zona pellucida (ZP) proteins. The characterized vertebrate genes encoding ZP proteins have been classified into six subfamilies, and exhibit low similarity to the ZP genes characterized in certain invertebrates. The origin and evolution of the vertebrate ZP genes remain obscure. A search against 97 representative metazoan species revealed various numbers (ranging from three to 33) of different putative egg-coat ZP genes in all 47 vertebrates and several ZP genes in five invertebrate species, but no putative ZP gene was found in the other 45 species. Based on phylogenetic and synteny analyses, all vertebrate egg-coat ZP genes were classified into eight ZP gene subfamilies. Lineage- and species-specific gene duplications and gene losses occurred frequently and represented the main causes of the patchy distribution of the eight ZP gene subfamilies in vertebrates. Thorough phylogenetic analyses revealed that the vertebrate ZP genes could be traced to three independent origins but were not orthologues of the characterized invertebrate ZP genes. Our results suggested that vertebrate egg-coat ZP genes should be classified into eight subfamilies, and a putative evolutionary map is proposed. These findings would aid the functional and evolutionary analyses of these reproductive genes in vertebrates.

## Abstract (precis)

**Summary:** Phylogenetic and synteny analyses indicate that the vertebrate zona pellucida (ZP) genes encoding egg coat proteins can be classified into eight subfamilies, and the evolutionary origins of these genes are discussed.

## INTRODUCTION

Animal egg coats participate in species-specific sperm-egg recognition during the process of fertilization and protect the growth of oocytes, eggs and early-developing embryos (Conner et al., 2005; Litscher and Wassarman, 2014; Modig et al., 2007). Egg coats are called different names in different organisms, such as vitelline membranes in echinoderms, chorions in fish, vitelline envelopes (VEs) in birds, reptiles and amphibians, and zona pellucida (ZP) in mammals.

Egg coats are composed of different numbers of glycoproteins, which also have different names depending on the methods through which they were identified (Goudet et al., 2008). All such characterized glycoproteins possess a common ZP module with approximately 260 amino acids (Litscher and Wassarman, 2014; Wilburn and Swanson, 2017) and are thus referred to as ZP glycoproteins regardless of the species from which the egg envelope was isolated (Hedrick, 2008). The genes encoding these glycoproteins have been intensively studied in vertebrates (Conner and Hughes, 2003; Li et al., 2011; Monné et al., 2006; Sano et al., 2013, 2010; Smith et al., 2005; Yue et al., 2014) and organized into six ZP gene subfamilies using an inconsistent nomenclature system (ZP1, ZP2 or ZPA, ZP3 or ZPC, ZP4 or ZPB, ZPD and ZPAX) (Claw and Swanson, 2012; Goudet et al., 2008; Spargo and Hope, 2003). The systematic distribution of ZP genes in vertebrates was recently reviewed (Litscher and Wassarman, 2014; Shu et al., 2015; Wu et al., 2018), although the evolutionary relationships among these ZP gene subfamilies remain unresolved. Based on gene structure and organization and synteny analyses, the ZP genes in vertebrates are thought to have been derived from a common ancestral gene that generated the ZP3 gene subfamily in one branch and the ZPD, ZPAX, ZP2, ZP1 and ZP4 gene subfamilies on the other branch through an ancient gene duplication event (Claw and Swanson, 2012; Litscher and Wassarman, 2014; Spargo and Hope, 2003). However, ZPX, which is mainly found in invertebrates, might be the result of a more ancient duplication event, as determined through a phylogenetic analysis (Wong and Wessel, 2006). Moreover, cloned ZP genes in *Xenopus laevis* (such as ZPY) have not yet been included in these analyses (Hedrick, 2008).

Interestingly, ZP proteins and their corresponding coding genes have also been characterized in cephalochordates (*Branchiostoma belcheri*) (Xu et al., 2012), urochordates (*Ciona savignyi* and *Ciona intestinalis*) (Kürn et al., 2007) and the archeogastropod abalone (*Haliotis spp.*) (Aagaard et al., 2010, 2006). These reports suggest an invertebrate origin for vertebrate egg-coat ZP genes (Xu et al., 2012), although a systematic phylogenetic analysis including both invertebrate and vertebrate egg-coat ZPs has not yet been conducted. Therefore, the origin and evolution of the vertebrate egg-coat ZP genes and the evolutionary relationships between the vertebrate and invertebrate egg-coat ZP genes have yet to be resolved.

Genomic data of diverse metazoans are becoming increasingly available, and this accumulation of data provides excellent opportunities for addressing the evolution of the egg-coat ZP genes. In the present study, a comprehensive taxa-wide investigation of egg-coat ZP genes was implemented to analyse their phylogenetic distributions in metazoans. Moreover, phylogenetic and synteny analyses were conducted to explore the origin and evolution of egg-coat ZP genes. Our results showed that vertebrate egg-coat ZP genes should be classified into eight subfamilies contained within the last common ancestor of vertebrates. In addition, these subfamilies could be further traced back to three ancestral ZP genes during the evolution of early vertebrates, but these three ancestral vertebrate ZP genes do not appear to have been directly inherited from the few invertebrate egg-coat ZP genes. This work provides a basis for studying these reproductive genes in different disciplines and performing functional analyses.

## RESULTS

### Phylogenetic distribution of egg-coat ZP genes and modification of the ZP gene subfamily definition

After performing the hmm searches and additional confirmations, a total of 446 ZP genes (smart00241: ZP module) were found in the genome of the 52 investigated representative species, which included 47 vertebrates and five invertebrates. All the identified ZP genes (with accession numbers, characteristic domains, genomic data sources and taxonomic information of the studied species) are listed in Table S1. The obtained putative ZP genes are non-redundant, and even the 21 ZP genes from four species that showed high identities (no less than 93%) with each other did not appear to be products of the same genomic locus (Table S2). The number of ZP genes found in each species ranged from three in mammals to 33 in Actinopterygii (Table 1), and our search did not find any putative ZP gene in the other 45 species.

**Table 1.**
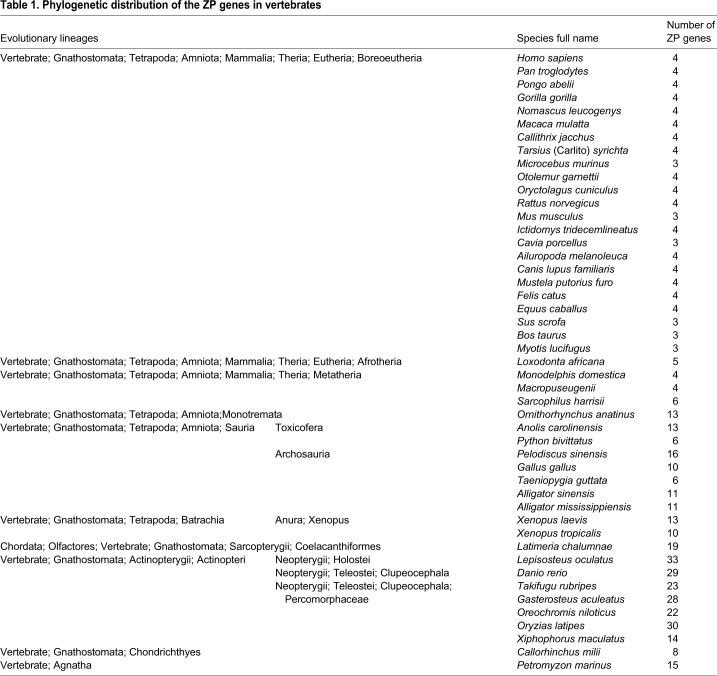
**Phylogenetic distribution of the ZP genes in vertebrates**

**Fig. 1.**
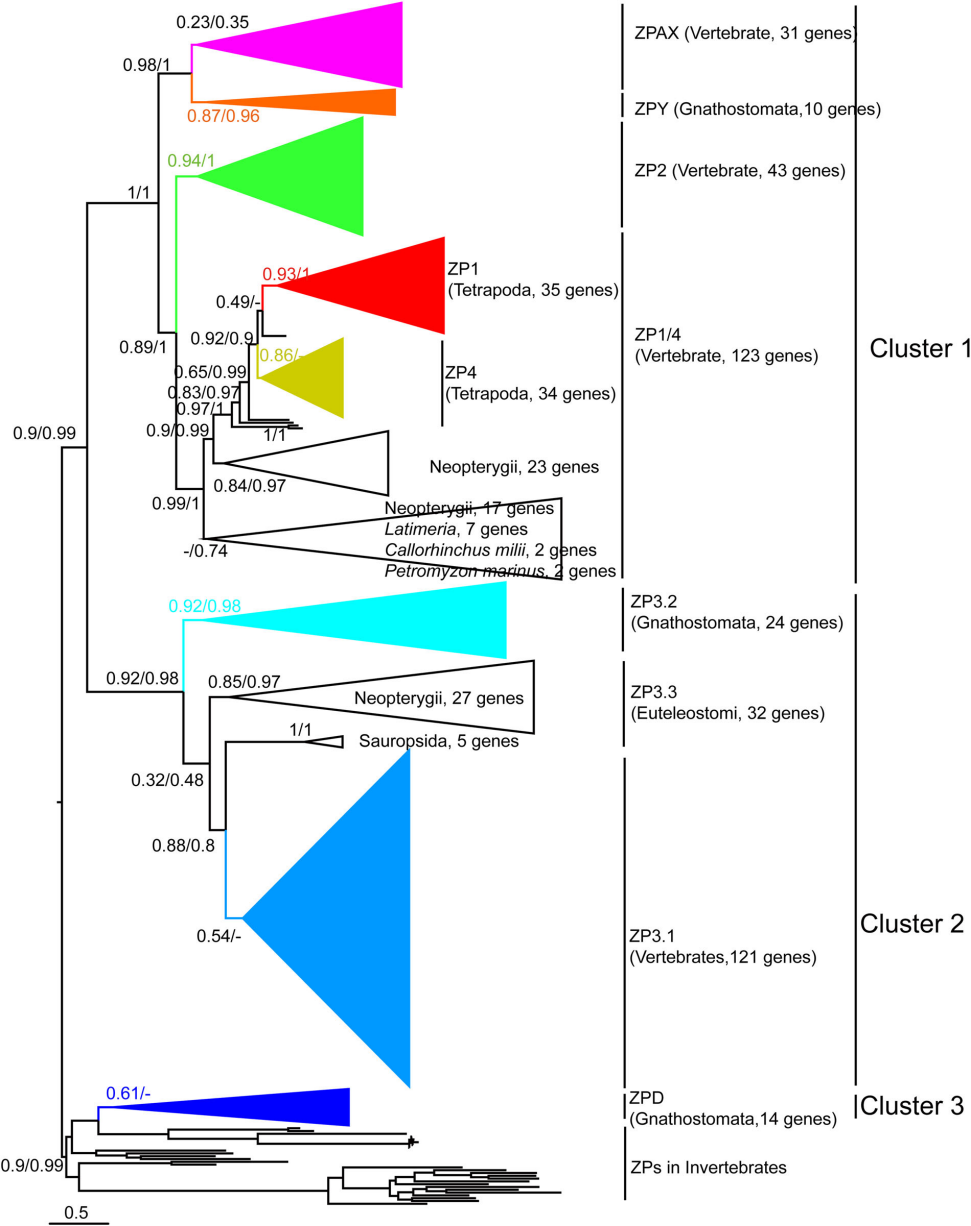
**Phylogenetic tree of the egg-coat ZP genes in vertebrates and invertebrates.** The ML tree was obtained based on 233 amino acid sites using FastTree v2.1.9 with the LG+CAT model. The statistical support (local support values for FastTree, aBayes values for PhyML) is indicated on the nodes, and ‘-’ indicates the absence of statistical support and/or incongruence between different ML methods. The support values of some nodes on Cluster 3 are not shown due to space limitation; they are shown in Fig. S1. The tree is arbitrarily rooted on the midpoint. The scale bar indicate the average number of amino acid substitutions per site.

Phylogenetic analyses of these characterized putative ZP genes were performed to evaluate the definition of ZP gene subfamilies in vertebrates and explore the distributions of ZP members in each representative vertebrate species. A total of 410 ZP module regions (SMART00241, more than 130 amino acids) were obtained and aligned. To explore the potential origin of these identified vertebrate ZP genes, previously characterized ZP genes of invertebrates (Aagaard et al., 2006; Kürn et al., 2007) as well as those characterized in this work, were included in the phylogenetic analyses. The two phylogenetic trees reconstructed using FastTree and PhyML showed similar topologies, and the ML tree (Fig. 1; Fig. S1) reconstructed using FastTree is shown here. We found six clades that were consistently recovered with the six previously reported ZP gene subfamilies (ZP1, ZP2, ZP3, ZP4, ZPD and ZPAX) (Fig. 1; Fig. S1). Significant differences were found between our phylogenetic tree and previously reported trees. First, the recovered ZP1 and ZP4 clades only consisted of homologues of Tetrapoda, and homologues of other vertebrates (Neopterygii, Coelacanth, Chondrichthyes and Agnatha) were recovered as outgroups. Interestingly, one small clade that consisted of *Xenopus* ZP genes was recovered, and this clade clustered with ZP1 with a consistently high support value (Fig. S1). Considering the phylogenetic topology and the common domain organization (Trefoil domain and ZP module) in the homologues of these clades, the ZP genes in these clades were collectively named the ZP1/4 gene subfamily. Second, one clade that was recovered with a high support value included ZPY of *Xenopus laevis* and ZP homologues of Neopterygii and Amniota was thus named the ZPY gene subfamily. Third, all known ZP3 genes were recovered in one big clade that could be further classified into several subclades. One of these subclades was recovered with the characterized ZP3 of all vertebrate lineages and another subclade was recovered with the annotated ZP3.2 of *Xenopus laevis* and other ZP homologues; thus, these subclades were named the ZP3.1 and ZP3.2 subfamilies, respectively. Additionally, two other separate monophyletic clades were recovered with homologues of Neopterygii and Sauria, and they were collectively named as the ZP3.3 gene subfamily. This putative ZP3.3 gene subfamily was further corroborated by the following phylogenetic analyses when fastest evolving sites were removed as described below.

**Fig. 2.**
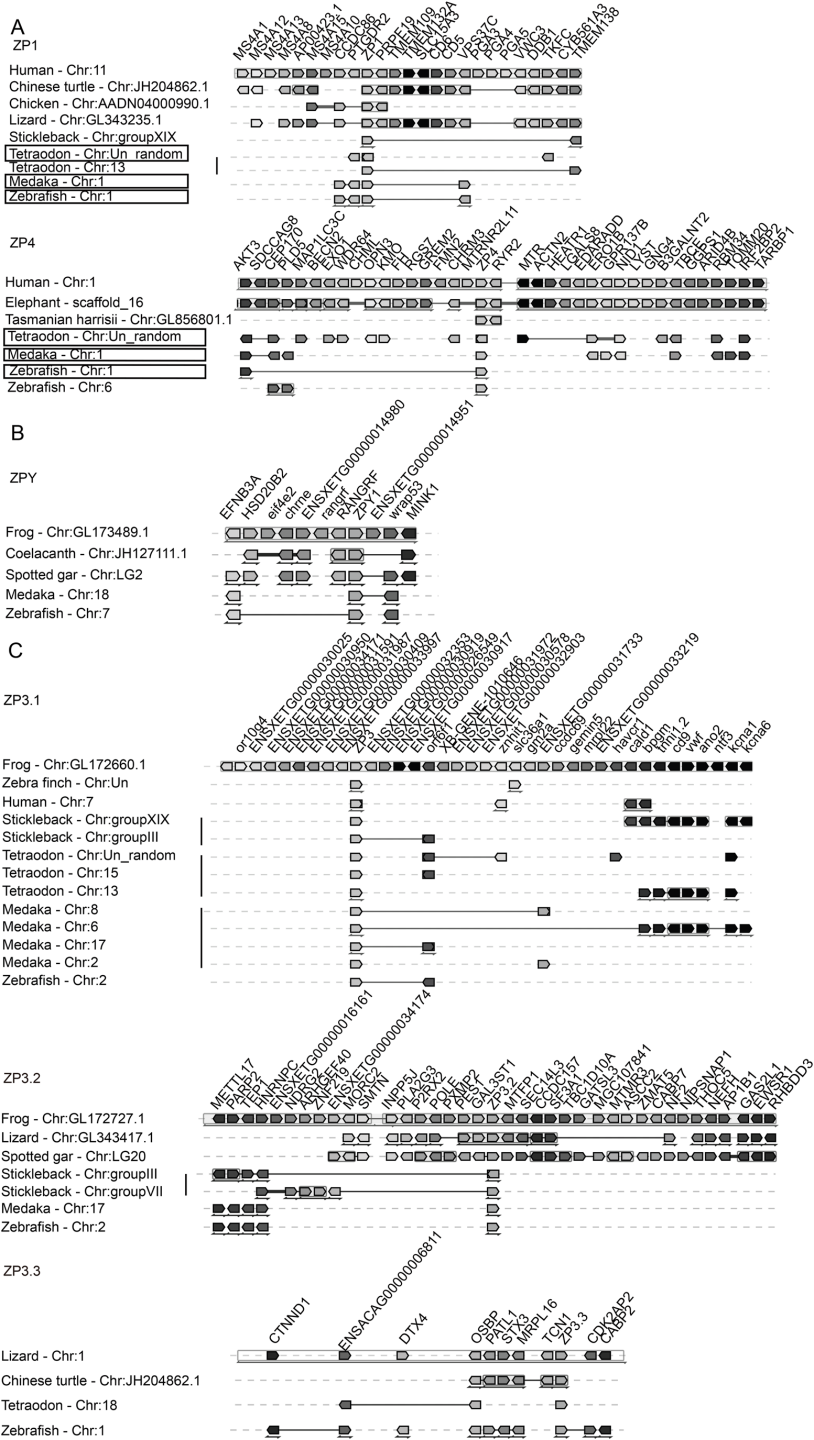
**Synteny of ZP gene subfamilies analysed using Genomicus.** (A) ZP1/4 gene subfamily, (B) ZPY gene subfamily and (C) ZP3.1, ZP3.2 and ZP3.3 gene subfamilies. The genes are indicated by block arrows that show their position and orientation in the genome and orthologous genes shared by species are shown in the same column. Teleosts possessing a conserved synteny map with both ZP1 and ZP4 of *Homo* are shown in boxes.

We conducted micro-synteny analyses of the above-mentioned ZP gene subfamilies to corroborate the results from the phylogenetic analyses. The conserved genomic neighbourhood surroundings of the four ZP gene subfamilies (ZP1/4, ZPY, ZP3.1 and ZP3.2) proposed in this study were detected (Fig. 2). Using the ZP1 and ZP4 genes of *Homo sapiens* as representatives, conserved synteny structures were found among the ZP1 and ZP4 genes of Tetrapoda and the ZP gene homologues of Neopterygii (Fig. 2A), indicating that ZP1 and ZP4 could have resulted from a Tetrapoda-specific gene duplication event. When *Xenopus tropicalis* was used as the reference species, conserved synteny of the ZP1 genes was also found among *Xenopus tropicalis* and the ZP1 genes of other species (Fig. S2A), which further corroborates the above-mentioned phylogenetic topologies. Although conserved synteny of the ZP4 gene was not found between *Homo* and Sauria, conserved synteny was found among *Gallus*, *Gorilla* and teleosts when *Gallus* was used as the reference species (Fig. S2). Conserved synteny structures were also found among the species of the two subclades that consisted of Neopterygii and Sauria (Fig. 2C), which confirmed a common origin of the species for the genes of these two subclades and thus these were classified as belonging to the ZP3.3 gene subfamily (Fig. 2C). Similarly, conserved synteny structures for the other three ZP gene subfamilies (ZP2, ZPAX and ZPD) were also found among different species (Fig. S2A).

Altogether, the results obtained in this study indicate that the previously proposed six ZP gene subfamilies should be reclassified into the following eight vertebrate egg-coat ZP gene subfamilies: the ZP1/4 gene subfamily, which includes ZP1 and ZP4 in Tetrapoda and ZP homologues of Neopterygii and other representative species; four newly proposed ZP gene subfamilies (ZPY, ZP3.1, ZP3.2 and ZP3.3 gene subfamilies), and three other ZP gene subfamilies (ZP2, ZPAX and ZPD) that were previously proposed. These eight ZP gene subfamilies can be further grouped into the following three large clusters: Cluster 1 consists of the ZP1/4, ZP2, ZPAX and ZPY gene subfamilies, Cluster 2 consists of the ZP3.1, ZP3.2 and ZP3.3 gene subfamilies and Cluster 3 consists of only the ZPD gene subfamily. All putative ZP genes of invertebrates were recovered as an outgroup of the ZPD clade but were not clustered with other vertebrate ZP gene subfamilies when the phylogenetic tree was rooted on the midpoint (Fig. 1; Fig. S1), indicating that the ZPD gene subfamily is not closely related with the other seven vertebrate ZP genes. Similar phylogenetic topologies were recovered even when the fastest evolving sites were removed, although the internal relationships of these seven ZP gene subfamilies could not be well resolved (Fig. S3). To explore the stability of these seven ZP subfamilies, the sequences of Clusters 1 and 2 were extracted and aligned, and were then separately subjected to phylogenetic analyses after excluding those fastest evolving sites. These ZP gene subfamilies (ZP1/4, ZP2, ZPAX, ZPY, ZP3.1, ZP3.2 and ZP3.3) were recovered as separate monophyletic clades (Figs S4 and S5).

**Fig. 3.**
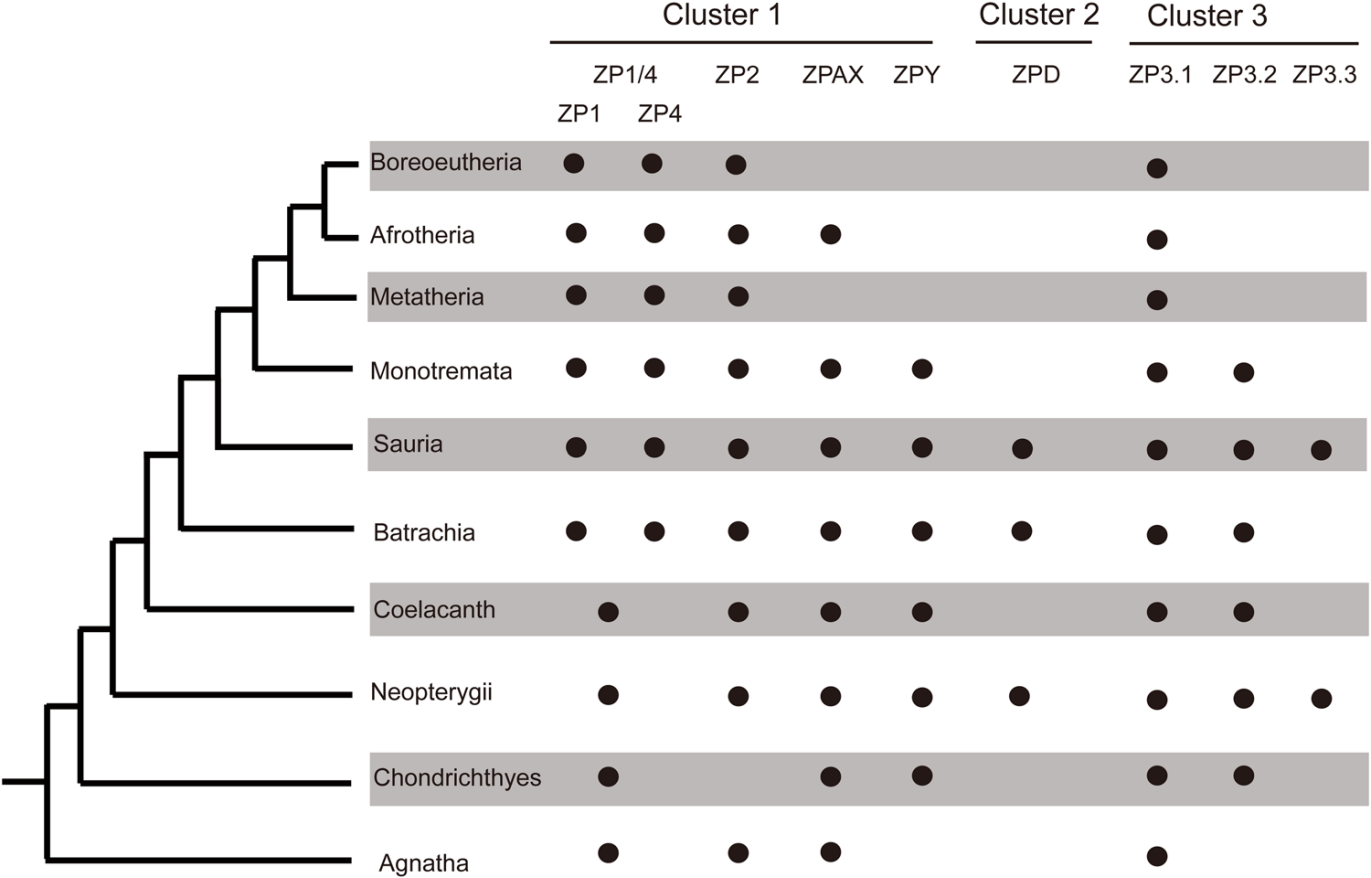
**Phylogenetic distributions of the eight ZP gene subfamilies in the main vertebrate lineages based on the phylogenetic analysis of all egg-coat ZP genes shown in** **Fig. 1****.** The names of the ZP gene subfamilies are presented at the top and the vertebrate lineages are presented on the left. A black dot indicates that the gene is present, whereas a blank space indicates that the gene is absent.

Based on the obtained phylogenetic topology, the eight ZP gene subfamilies showed unequal distribution patterns in different vertebrate lineages. The ZP1/4 and ZP3.1 gene subfamilies were widely distributed; the ZP2, ZPAX, ZPY and ZP3.2 gene subfamilies were absent in several lineages; the ZPD gene subfamily was only found in three lineages; and the ZP3.3 gene subfamilies were only found in two lineages. The detailed phylogenetic distributions of each of these eight ZP gene subfamilies are summarized in Fig. 3.

### Frequent gene duplication and gene loss during the evolution of each egg-coat ZP gene subfamily

Eight ZP gene subfamilies contained more than one member in many of the investigated vertebrate species (Table S1), which suggests that gene duplications of the ZP genes occurred during the evolution of vertebrates. The phylogenetic and synteny analyses revealed the gene duplication and gene loss events that occurred at different evolutionary stages.

(1) The eight ZP gene subfamilies were recovered into three clusters, and the recovery of putative ZP genes of lamprey (*Petromyzon marinus*), the earliest diverging extant vertebrate, in the ZP1/4, ZP2, ZPAX and ZP3.1 gene subfamilies indicated that the eight ZP gene subfamilies were obtained via ancient gene duplication events in the last common ancestor of vertebrate from three ancestral ZP genes. (2) Lineage-specific gene duplication frequently occurred in certain ZP gene subfamilies: ZP1 and ZP4 genes were obtained through Tetrapoda-specific gene duplication, and the ZP1/4 homologues of Neopterygii experienced Percomorphaceae-specific gene duplication (Fig. S1). For the ZPAX, ZP3.1, ZP3.2 and ZP3.3 gene subfamilies, ZP homologues of Neopterygii could also be further recovered into different subclades, which indicated that these were obtained through Neopterygii-, Clupeocephala-specific and/or Percomorphaceae-specific gene duplication events (Fig. S1). Noticeably, three ZP genes of three species (*Takifugu rubripes*, *Alligator sinensis* and *Alligator mississippiensis*) possessed two ZP modules (Table S1 and Fig. S6A), and these modules were recovered in separate branches, which indicated that they were obtained through recent gene duplication events that occurred in different lineages. (3) A number of ZP gene homologues in certain species were clustered together on the phylogenetic tree and were confirmed to be tandem repeats on their chromosome, which suggested that they were obtained through species-specific gene duplication. These homologues include the six ZP1/4 gene homologues of *Danio rerio* (Figs S1 and S6B).

Gene loss events also occurred frequently. Only a few homologues of mammal ZP genes were recovered in the ZPAX, ZPY, ZP3.2 and ZP3.3 gene subfamilies, which suggested that massive gene loss events occurred in mammals during evolution (Fig. 3; Fig. S1). To detect the presence of pseudogenes of the three newly defined ZP gene subfamilies during evolution, the amino acid sequences of the ZPY (XP_008116403.1), ZP3.2 (XP_008118026.2) and ZP3.3 (XP_008108877.1) genes of *Anolis carolinensis* were used as queries to search against the genomes of the Tetrapoda species in which these three ZP genes were not found by tBLASTn analyses. Evidence of pseudogenes was found in certain species (Table S3). Significant alignments were found, and stop codon(s) were observed, which suggested the presence of the ZPY pseudogene in *Taeniopygia guttata*, *Alligator sinensis* and *Alligator mississippiensis*, the ZP3.2 pseudogene in *Ailuropoda melanoleuca*, *Felis catus* and *Monodelphis domestica*, and the ZP3.3 pseudogene in *Python bivittatus*. Additionally, certain segments that showed high sequence similarity to the ZP3.2 gene were also found on the genome of *Equus caballus*, *Taeniopygia guttata* and *Python bivittatus*, although no stop codon was found. All these results are summarized in Fig. S7.

### Evolutionary origin of egg-coat ZP genes in vertebrates

**Fig. 4.**
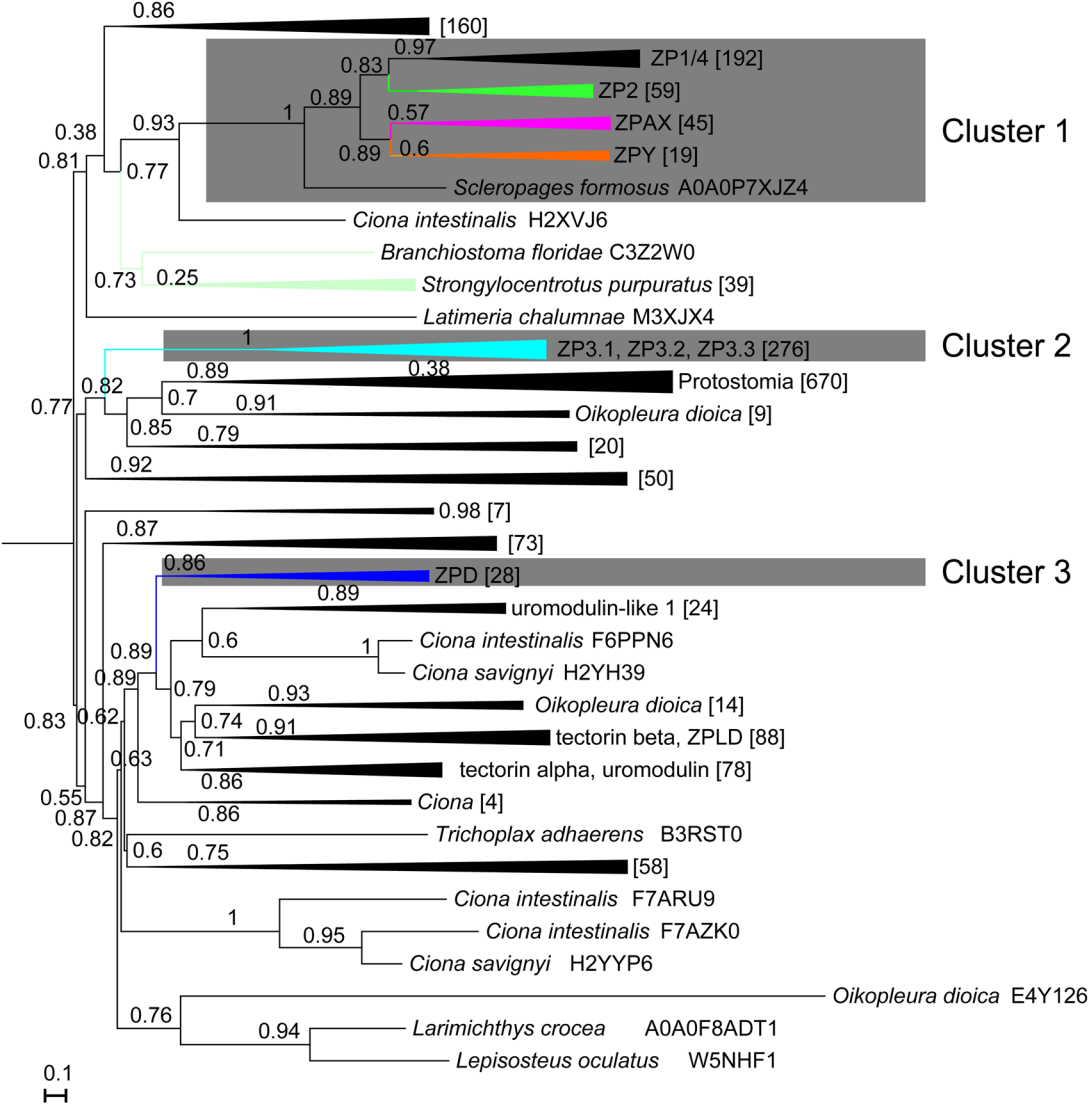
**Phylogenetic tree of the egg-coat ZP genes and representative ZP module-containing genes retrieved from the PFAM database.** The ML tree was obtained based on 160 amino acid sites using Fasttree v2.1.9 with the LG+CAT model. The statistical support (local support values for FastTree) is indicated on the nodes. The tree is arbitrarily rooted on the midpoint. The scale bars indicate the average number of amino acid substitutions per site.

The recovery of *Petromyzon marinus*, the earliest diverging extant vertebrate lineage, in four clades (ZP1/4, ZP2, ZPAX and ZP3.1) of the eight ZP gene subfamilies suggested that these four ZP gene subfamilies might already be present in the last common ancestor of vertebrates. Although these eight ZP gene subfamilies could be further clustered into three large clades (named Clusters 1, 2 and 3) based on the obtained phylogenetic tree (Fig. 1; Fig. S1), the relationships among the vertebrate ZP genes remain obscure because only a limited number of invertebrate ZP genes were included. When ZPD of *Xenopus laevis* (NP_001081431.1) was used as the query for the search against the Refseq_proteins database of the National Center for Biotechnology Information (NCBI), the top hits were uromodulin-like genes, followed by pancreatic secretory granule membrane major glycoprotein (GP2), tectorin alpha genes and others. Thus, we subsequently conducted two separate phylogenetic analyses to clarify the potential evolutionary relationships among these eight identified ZP gene subfamilies. First, all ZP genes identified in this work and the representative genes of the ZP module-containing genes (the seed ZP genes used to build hmmprofile in the Pfam database, 1926 sequences in total) were aligned and subjected to phylogenetic analyses using the FastTree and PhyML methods. In the obtained phylogenetic tree (Fig. 4; Fig. S8), three large clades (Clusters 1, 2 and 3) were recovered, and these were clustered into eight ZP gene subfamilies (ZP1/4, ZP2, ZPAX, ZPY, ZPD, ZP3.1, ZP3.2 and ZP3.3), which are consistent with those shown in Fig. 1. Furthermore, Cluster 3 was consistently grouped with uromodulin-like, tectorin beta, Zona pellucida-like domain-containing protein 1 (ZPLD), tectorin alpha and GP2 in both trees reconstructed by using the two methods (Fig. 4; Fig. S8); however, the sistergroups of Cluster 1 and 2 showed slight differences between the two phylogenetic trees reconstructed using the two methods (Fig. 4; Fig. S8). In all cases, these three clusters were grouped separately (Fig. 4; Fig. S8). Second, using the ZPD of *Xenopus laevis* (NP_001081431.1) as the query, the top 500 hits found by searching against the NCBI and top three hits found in the genome of *Petromyzon marinus* on ENSEMBL were extracted, aligned and subjected to a phylogenetic analysis. In the obtained phylogenetic tree (Fig. S9), one monophyletic clade, which consisted of the ZPD gene of *Xenopus* and the uromodulin-like genes of other reptiles, teleosts and Chondrichthyes, was recovered, and one clade consisting of alpha-tectorin-like and/or GP2-like genes was recovered as its sister group. Two ZP homologues of *Petromyzon marinus* were recovered as the outgroup of vertebrate tectorin alpha, and then grouped with GP2 and ZP homologues of Cephalochordata were recovered as an outgroup of these two clades.

**Fig. 5.**
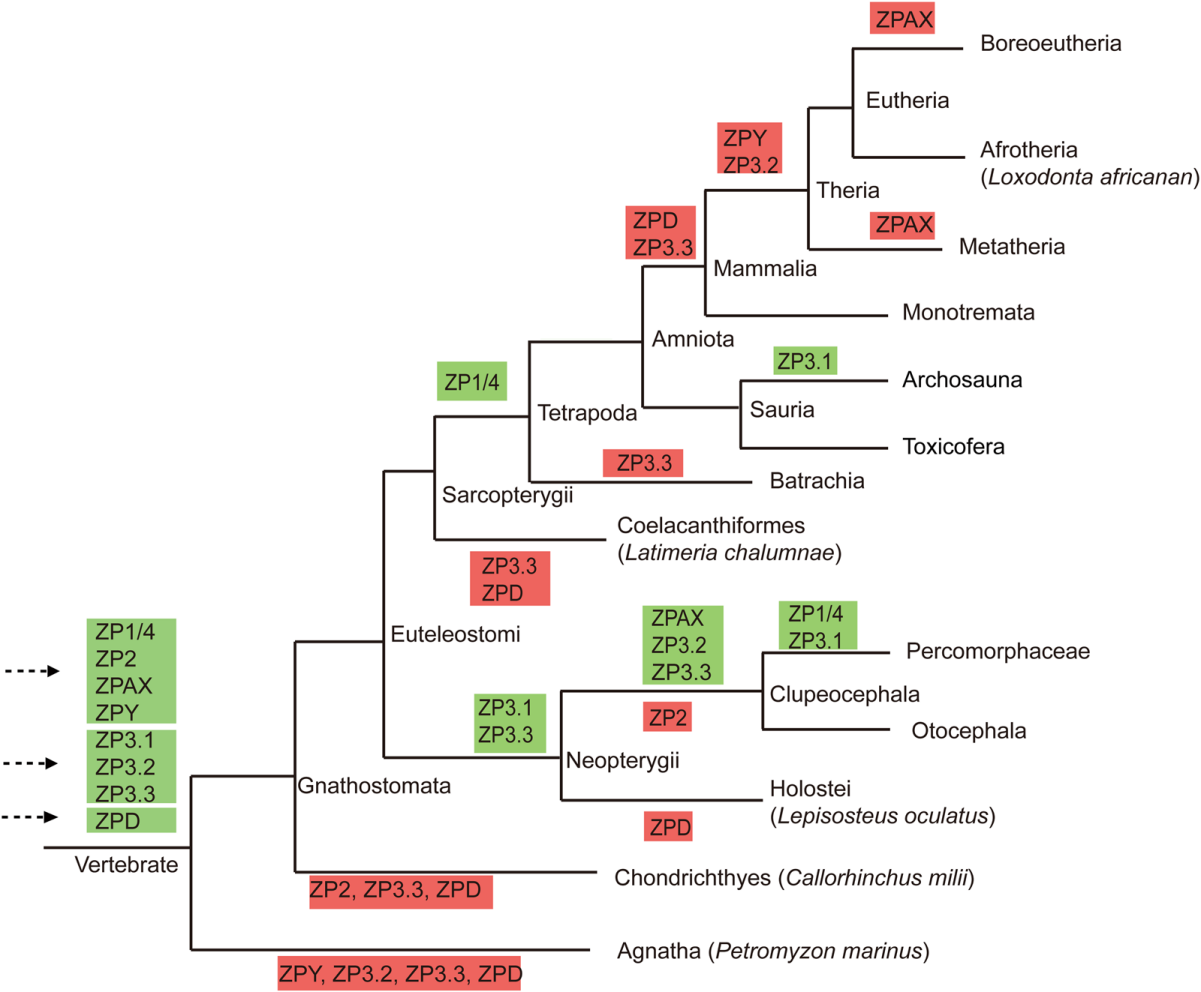
**Putative evolutionary map of egg-coat ZP genes in vertebrates.** The phylogenetic relationships among the studied lineages are shown (based on the NCBI taxonomy browser). Green and red boxes indicate gene duplications and gene losses that occurred in vertebrate lineages, respectively, and gene duplication and loss events that occurred in species are not shown.

Based on all these results, a putative evolutionary map of eight vertebrate ZP gene subfamilies was proposed (Fig. 5). Briefly, three separate ancestral ZP genes were presented in the last common ancestor of vertebrate and quickly expanded into eight gene subfamilies, and then these eight members were experienced massive lineage- and/or species-specific gene duplication/loss events, which resulted in the patchy and complicated distribution pattern of ZP genes in vertebrates.

## DISCUSSION

### Modification of the nomenclature of vertebrate egg-coat ZP gene subfamilies and their patchy phylogenetic distributions

The egg-coat ZP proteins characterized in vertebrates are considered a group of conserved proteins (Litscher and Wassarman, 2014). Based on the high divergence of different ZP genes, which are thought to be rapidly evolving reproduction genes (Aagaard et al., 2006; Swanson and Vacquier, 2002), and the presence of many other ZP module-containing proteins, as was recently reported (Jovine et al., 2005; Plaza et al., 2010), the precise identification of egg-coat ZP genes is a critical step in the study of the evolution of this reproductive gene family. Using the custom hmm profile built from the characterized ZPs, 446 ZP homologues were detected in 52 species and no additional ZP module-containing genes were found, indicating the specificity of our hmm profile and greatly reducing the subsequent computational burden. The sequence identities of most of the ZP genes (425 of 446) identified in each species were lower than 93%, which suggested that they do not appear to be falsely duplicated genes caused by genome misassembly errors (Denton et al., 2014; Kelley and Salzberg, 2010; Zhang and Backström, 2014). Compared with the six ZP gene subfamilies previously proposed based on phylogenetic analyses of limited ZP genes (Goudet et al., 2008), this study applied the results of phylogenetic and synteny analyses to classify the identified putative vertebrate egg-coat ZP genes into eight ZP gene subfamilies (ZP1/4, ZP2, ZPAX, ZPY, ZPD, ZP3.1, ZP3.2 and ZP3.3) according to the nomenclature used by Goudet et al. (2008) with minor revisions.

Great variations in the characterized ZP gene numbers were found among different species, particularly between Actinopterygii and mammals (Table 1). These notable variations in the ZP gene numbers among different lineages could be explained by lineage-specific gene duplications, such as the Tetrapoda-specific gene duplication of ZP1/4 and consecutive Neopterygii- and Clupeocephala-specific gene duplications in most of the proposed ZP gene subfamilies (e.g. ZP1/4, ZPAX, ZP3.1, ZP3.2 and ZP3.3) (Fig. 5; Fig. S1) and tandem duplications occurred in some species or lineages, such as those found for ZP1/4 in *Danio rerio*, *Alligator sinensis* and *Alligator mississippiensis* (Figs S1 and S6A,B). In fact, lineage- and species-specific gene duplications have been previously revealed in Teleostei (Conner and Hughes, 2003; Sano et al., 2013, 2010) and notothenioids (Cao et al., 2016). Such duplicated ZP genes appear to be responsible for their tissue-specific expression and/or the acquisition of novel physiological functions required for adapting to specific environments (Shu et al., 2015). In addition, extensive gene loss events that occurred in different vertebrate lineages might be a cause of the patchy distribution pattern of these eight ZP gene subfamilies. For example, the loss of most ZP gene subfamilies occurred repeatedly in mammals (Figs 3 and 5). This gene loss has been explained as a progressive process during the evolution of vertebrates due to the presence of pseudogenes of ZP1, ZP4 and ZPAX in certain mammals (Goudet et al., 2008; Moros-Nicolás et al., 2017). Similarly, putative pseudogenes of ZPY, ZP3.2 and ZP3.3 were also found in different species (Table S3; Fig. S7). Such an absence might also be caused by unfilled gaps and/or incorrect annotation of the genome. The absence of the ZP2 gene subfamily in most species of Neopterygii (with the exception of *Lepisosteus oculatus*) appears to have been caused by unfilled gaps in their genomes (Fig. S2B). No homologue of ZPY was detected in the genome of *Loxodonta africana* downloaded from the NCBI; however, one similar sequence (ENSLAFG00000030361) was found on the ENSEMBL database, and a subsequent synteny analysis confirmed that this sequence was a ZPY homologue (Fig. S2A). Such inconsistencies would be greatly reduced by more accurate and updated genome annotation in the future. All these facts suggest that the ZP genes were progressively lost during the evolution of vertebrates.

The extensive gene duplication and progressive gene loss events that occurred during the evolution of vertebrates indicated that the ZP gene is a rapidly evolving reproductive gene and the rapid evolution of this gene results in the notable variation in ZP members and the patchy distributions of ZP gene subfamilies in different vertebrate species. Duplicated ZP genes might provide selective targets for the adaptation to different ecological environments (Cao et al., 2016) and the provision of species-specific barriers during fertilization (Bianchi and Wright, 2015; Claw and Swanson, 2012).

### Three independent evolutionary origins of the vertebrate egg-coat ZP gene subfamilies

The six previously proposed ZP gene subfamilies are thought to have been obtained through ancient gene duplication events, but their evolutionary relationships remain obscure (Claw and Swanson, 2012; Goudet et al., 2008; Spargo and Hope, 2003; Wong and Wessel, 2006). A number of egg-coat ZP genes characterized in the basal chordate and the archeogastropod abalone suggest an invertebrate origin for the vertebrate egg-coat ZP genes (Aagaard et al., 2010, 2006; Kürn et al., 2007; Xu et al., 2012); however, previously conducted phylogenetic analyses of ZP genes did not include invertebrate egg-coat ZP homologues (Goudet et al., 2008; Spargo and Hope, 2003). Therefore, the evolutionary relationships among the vertebrate and invertebrate egg-coat ZP genes require illustration.

Considering the consistent phylogenetic topology obtained using different phylogenetic analysis methods and the inclusion of *Petromyzon marinus* in four ZP gene subfamilies (ZP1/4, ZP2, ZPAX and ZP3.1), we presumed that the ZP1/4, ZP2, ZPAX and ZP3.1 subfamilies as well as the ZPY, ZP3.2 and ZP3.3 subfamilies might have already emerged in the vertebrate ancestor. The robust recovery of two large monophyletic clades (Clusters 1 and 2) consisting of seven clades corresponding to seven ZP gene subfamilies suggested that these seven ZP gene subfamilies could be traced back to two ancestral ZP genes during the evolution of early vertebrates. To explore the evolutionary origins of the three clusters of ZP genes, representative ZP module-containing genes were also included in the phylogenetic analysis. Clusters 1, 2 and 3 were consistently recovered as separate clusters and were grouped into eight ZP gene subfamilies (ZP1/4, ZP2, ZPAX, ZPY, ZPD, ZP3.1, ZP3.2 and ZP3.3) in the phylogenetic trees (Fig. 4; Fig. S8). Moreover, the early diverging chordates (*Ciona intestinalis* and *Branchiostoma floridae*) were recovered in the sister group of Cluster 3 (ZPD) consistently in the ML trees obtained using two different methods (Fig. 4; Fig. S8), which suggested that the ZPD gene might have been obtained in the last common ancestor of Chordata. Similarly, in the phylogenetic tree reconstructed with ZPD genes and the top 500 hits retrieved from the NCBI database, the ZPD genes were recovered as a monophyletic clade and were then grouped with other ZP module-containing genes (Fig. S9), and ZP homologues of *Petromyzon marinus* and *Branchiostoma* were recovered as deep branches in another clade that consisted of alpha tectorin and GP2 (Fig. S9), which suggested that the ZPD gene might have also been derived from ancient gene duplication events that occurred in the last common ancestor of Chordata. Cluster1 and 2 were grouped with other ZP module-containing genes and with different ZP module-containing genes from different lineages in the ML trees obtained using FastTree (Fig. 4) and PhyML (Fig. S8), respectively. This inconsistency might be explained by the fact that the ZP genes in these two clusters showed marked differences from the other ZP module-containing genes, and the origins of these two clusters remain to be elucidated. In all cases, the three clusters were separated on both phylogenetic trees, which suggested they could be derived from three separate ancient gene duplication events. All these findings suggested that these eight ZP gene subfamilies could be traced back to three ancestral ZP genes, which appear to have been observed at least in the last common vertebrate ancestor. Overall, this work provides a basis for further physiological functional analyses and insights into the origin and evolution of these reproductive genes.

## MATERIALS AND METHODS

### Data sources

Representatives of 97 metazoan species belonging to nonbilaterians (Porifera, Ctenophora, Cnidaria and Placozoa), Lophotrochozoans (Mollusca, Annelida, Rotifera and Platyhelmintha), Ecdysozoans (Nematoda and Arthropoda) and Deuterostomes were selected. These 97 representative species were chosen mainly according to Vervoort et al. (2016) and meet the following two criteria: (1) the vertebrate species were selected if genomic data were available; (2) if genomic data from multiple species that were closely related (e.g. belonging to the same genus) were available, only one of these species was kept as a representative species in the analyses to reduce the computational burden. For example, only *Bos taurus* was used even though two other closely related species were also sequenced (*B. indicus* and *B. mutus*). Their genome data were mainly retrieved from NCBI, ENSEMBL and Vector databases (last accessed Oct. 2017; see Table S1).

### Identification of egg-coat ZP genes

To specifically and comprehensively identify putative vertebrate egg-coat ZP genes, 21 well-characterized ZP genes of *Homo sapiens*, *Xenopus laevis* and *Oryzias latipes* were retrieved from the UniProt and NCBI databases (Table S4), and a custom HMM profile was constructed based on multiple sequence alignment of the amino acid sequences of these 21 ZP genes using the hmmbuild tool within HMMER 3.0 (Eddy, 1998). The custom hmm profile was used to identify potential ZP genes in the genome data from the 97 representative species using the hmmsearch tool (E value cut-off of 0.001) (Eddy, 1998). Hit sequences were recognized as putative egg-coat ZP genes if they (1) allowed for the retrieval of egg-coat ZP genes as best hits when they were used as queries for a BLAST search against the genome of three species (*Homo sapiens*, *Xenopus laevis* and *Oryzias latipes*) and (2) contained the characterized ZP module (smart00241) and/or the ZP-like domain (PF00100) (Okumura et al., 2015; Plaza et al., 2010). The obtained hits were subjected to the batch CD-search tool (Marchler-Bauer and Bryant, 2004; Marchler-Bauer et al., 2011) to confirm the presence of the ZP module (smart00241) and/or the ZP-like domain (PF00100) and other domains, e.g. Trefoil (pfam00088) and PD (smart00018). To ensure that only one protein sequence was retrieved from each genomic locus, the corresponding gene IDs of all the hits were retrieved using the E-utilities (Sayers, 2010). The longest protein was selected and used for the following analyses. Moreover, the identified ZP genes of each species were compared separately using Muscle V3.8 (Edgar, 2004), and the protein sequences that showed more than 93% sequence identity with each other were further checked to determine whether they were the products of the same genomic locus.

### Multiple sequence alignments and phylogenetic analyses

Considering the presence of multiple different domains in the identified putative egg-coat ZP genes, only the characterized ZP modules (smart00241, more than 130 amino acids) identified by the batch CD-search were extracted using our custom Perl scripts and then aligned to the hmmprofile of the ZP module (PF00100) using the hmmaligntool of HMMER3.0 (Eddy, 1998). The other ZP modules (less than 130 amino acids) were discarded.

After removing poorly aligned columns using TrimAl (–gt option 0.75) (Capella-Gutierrez et al., 2009), the multiple sequence alignment was subjected to a phylogenetic analysis using FastTree v2.1.9 (Price et al., 2010) in the ‘slow and accurate’ mode (-spr 4 -mlacc 2 -slownni) with the LG+CAT model and using PhyML v3.1 (Guindon et al., 2010; Le and Gascuel, 2008) with the LG model plus four gamma distribution and invariant sites (LG+Γ4+I). The branch support for the ML tree obtained using PhyML was determined through a Bayesian-like transformation of aLRT (aBayes) (Anisimova et al., 2011). To assess the effect of rapidly evolving sites on tree topology, relative evolutionary rates were estimated using Tree Independent Generation of Evolutionary Rates (TIGER) v.1.02 (Cummins and McInerney, 2011). The alignment sites were assigned to 30 rate bins, the fastest evolving sites (BIN30) were excluded from further phylogenetic analyses and the remaining variant sites were subjected to phylogenetic analyses as mentioned above.

### Synteny comparisons

To further validate the topology of the obtained phylogenetic tree, a genomic synteny analysis was performed using Genomicus (version 88) (http://genomicus.biologie.ens.fr/genomicus-88.01/cgi-bin/search.pl) (Louis et al., 2015). Synteny maps for the genomic neighbourhoods surrounding each of the identified ZP gene subfamilies in human (*Homo sapiens*), chicken (*Gallus gallus*), anole lizard (*Anolis carolinensis*), western clawed frog (*Xenopus tropicalis*), medaka (*Oryzias latipes*), stickleback (*Gasterosteus aculeatus*), zebrafish (*Danio rerio*) and green spotted puffer (*Tetraodon nigroviridis*) were constructed with AlignView (Louis et al., 2013). If no conserved structure was found among these eight representative species, close relatives of these eight species with conserved synteny maps were selected as representative species. For example, zebra finch was included because no conserved synteny map for the ZP3.1 gene was found in *Gallus gallus*. The frog ZP gene was selected as a reference gene in the six proposed ZP gene subfamilies, and the ZP genes of human and lizard were selected as reference genes in the ZP1/4 and ZP3.3 gene subfamilies, respectively.

## Supplementary Material

Supplementary information
