# Supplementary material for: Evolution and multiple origins of zona pellucida genes in vertebrates
Source: Biol Open. 2018 Nov 15;7(11):bio036137. doi: 10.1242/bio.036137 (PMC6262864; doi:10.1242/bio.036137)
Supplement: Supplementary information [file biolopen-7-036137-s1.pdf]

Table S1. Characterized egg-coat ZP genes that belong to the eight ZP subfamilies in vertebrates. Zona_pellucida (PF00100, Zona pellucida-like domain), ZP (smart00241, Zona pellucida [ZP] domain), Trefoil (pfam00088, Trefoil [P-type] domain). (N), (C) and (NC) correspond to incompleteness at the N terminus, C terminus, or both the N and C termini, respectively. The ZP proteins used as representatives in the following phylogenetic analyses are shown in bold, and the redundant and/or incomplete ones are shown in italics.

Click here to Download Table S1

Table S2. Genome loci of the 21 ZP genes showing high similarity that were characterized in the four species.

Click here to Download Table S2

Table S3. Identification of pseudogenes of each ZP gene on the genome of Tetrapoda species. +, the presence of this gene; Pseudogene, the detection of segments with stop codon(s) and with high sequence similarity; High sequence similarity, the detection of segments without stop codons and with high sequence similarity; Not found, no pseudogene was found because either a genomic fragment was completely lost or the genome was not completely sequenced.

Click here to Download Table S3

Table S4. Egg-coat ZPs used as queries in this work.

Click here to Download Table S4

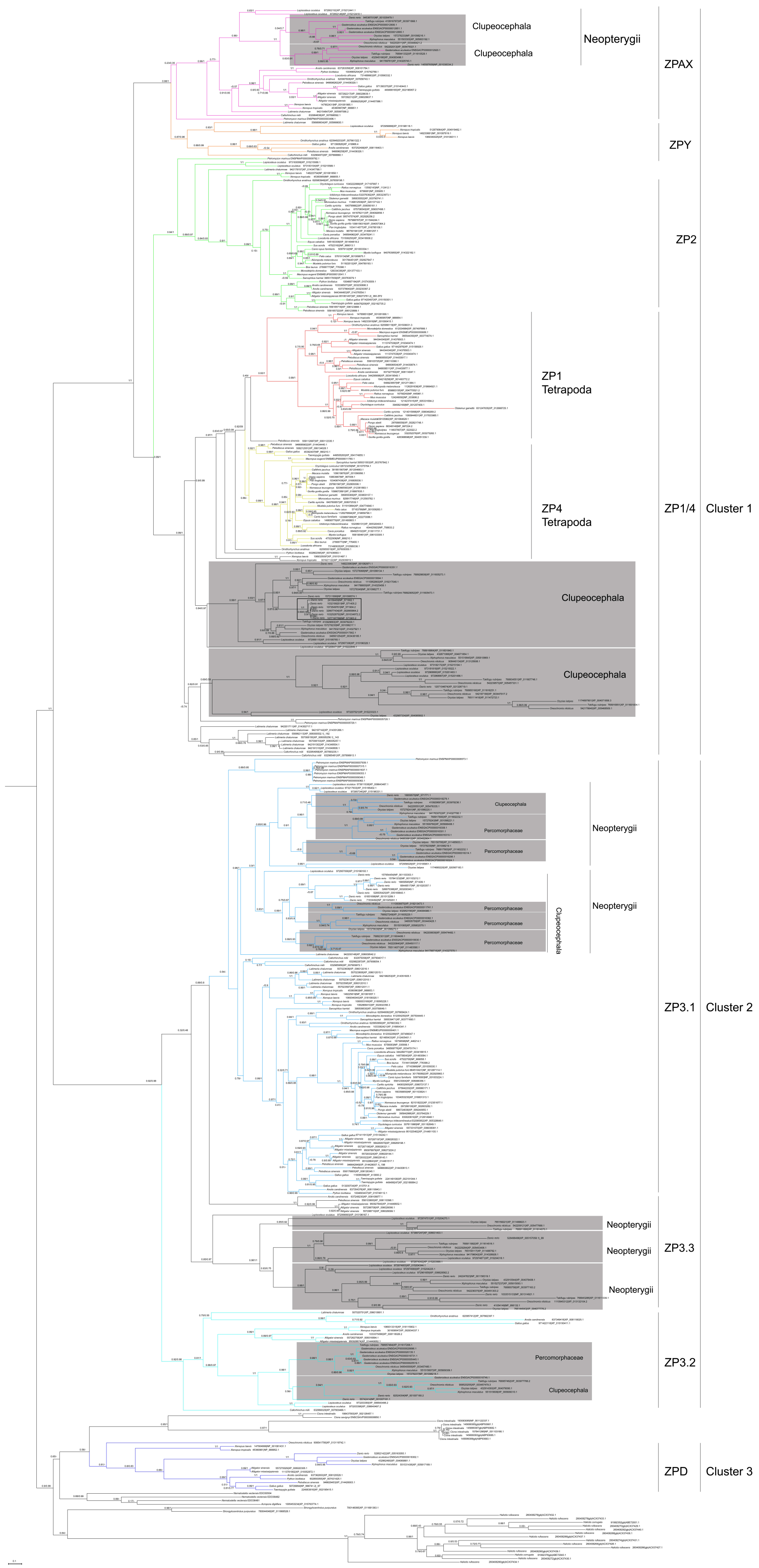

Clupeocephala
Clupeocephala
Neopterygii
ZPAX
ZPY
ZP2
ZP1
Tetrapoda
ZP4
Tetrapoda
ZP1/4
Cluster 1
Clupeocephala
Clupeocephala
Clupeocephala
Percomorphaceae
Percomorphaceae
Neopterygii
Percomorphaceae
Percomorphaceae
Percomorphaceae
Clupeocephala
Neopterygii
ZP3.1
Cluster 2
Neopterygii
Neopterygii
Neopterygii
ZP3.3
Percomorphaceae
Clupeocephala
ZP3.2
ZPD
Cluster 3

Fig. S1. Phylogeny of all identified vertebrate ZP genes. The phylogenetic tree was inferred based on an aligned amino acid dataset with 233 sites. Lineage-specific duplication events are shown in grey. The numbers shown in the nodes correspond to the support values obtained with FastTree and PhyML. The six characterized ZP1/4 homologs of *Danio rerio* obtained through tandem duplication are marked in box. The scale bar indicates the average number of amino acid substitutions per site.

Biology Open • Supplementary information

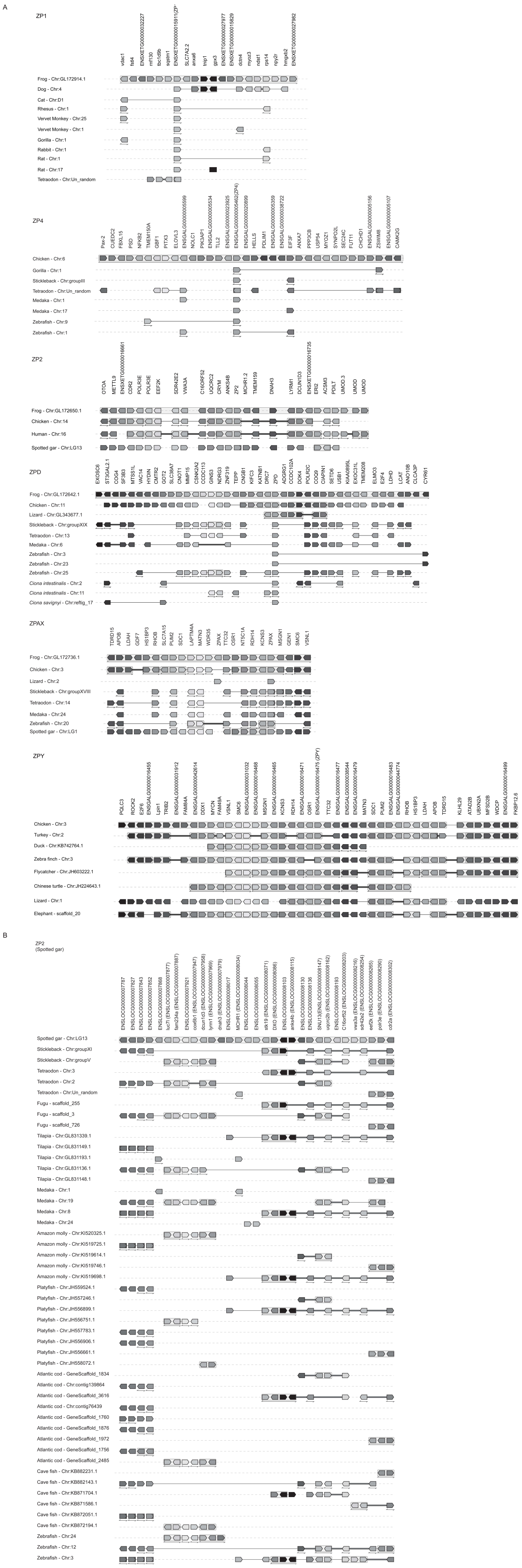

Biology Open • Supplementary information

Fig. S2. (A) Conserved synteny structures of the ZP1, ZP4, ZP2, ZPD, ZPAX, and ZPY gene subfamilies. The reference species are listed on the top. (B) ZP2 gene on the genome of *Lepisosteus oculatus* and rooted at the Neopterygii ancestor. The details are the same as those of Fig. 2.

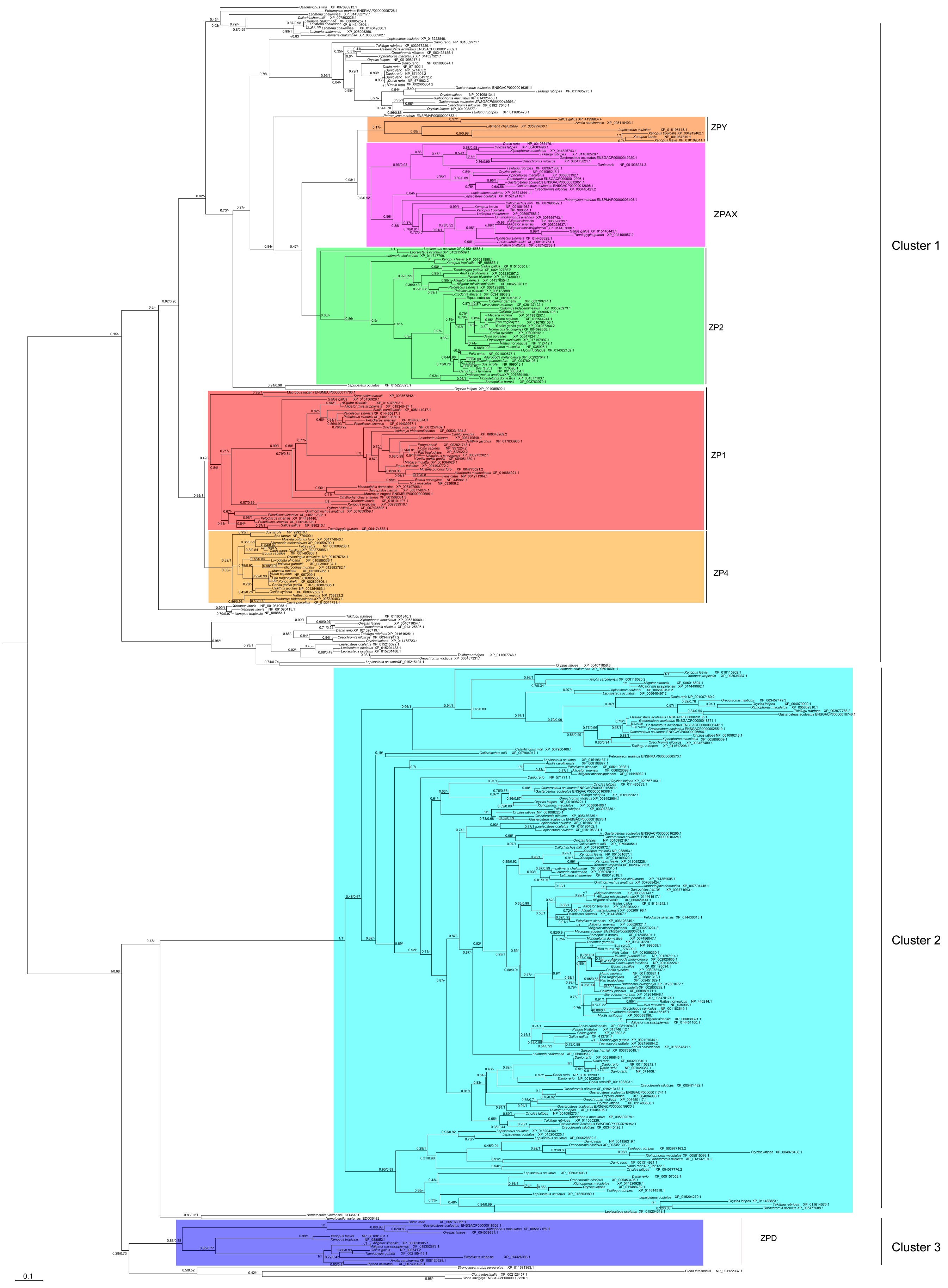
ZPY
ZPAX
ZP2
ZP1
ZP4
Cluster 1
Cluster 2
ZPD
Cluster 3
0.1

Fig. S3. Phylogeny of all identified vertebrate ZP genes after excluding the fastest-evolving sites. The phylogenetic tree was inferred based on an aligned amino acid dataset with 114 sites. The numbers shown in the nodes correspond to the support values obtained with FastTree and PhyML. The scale bar indicates the average number of amino acid substitutions per site.

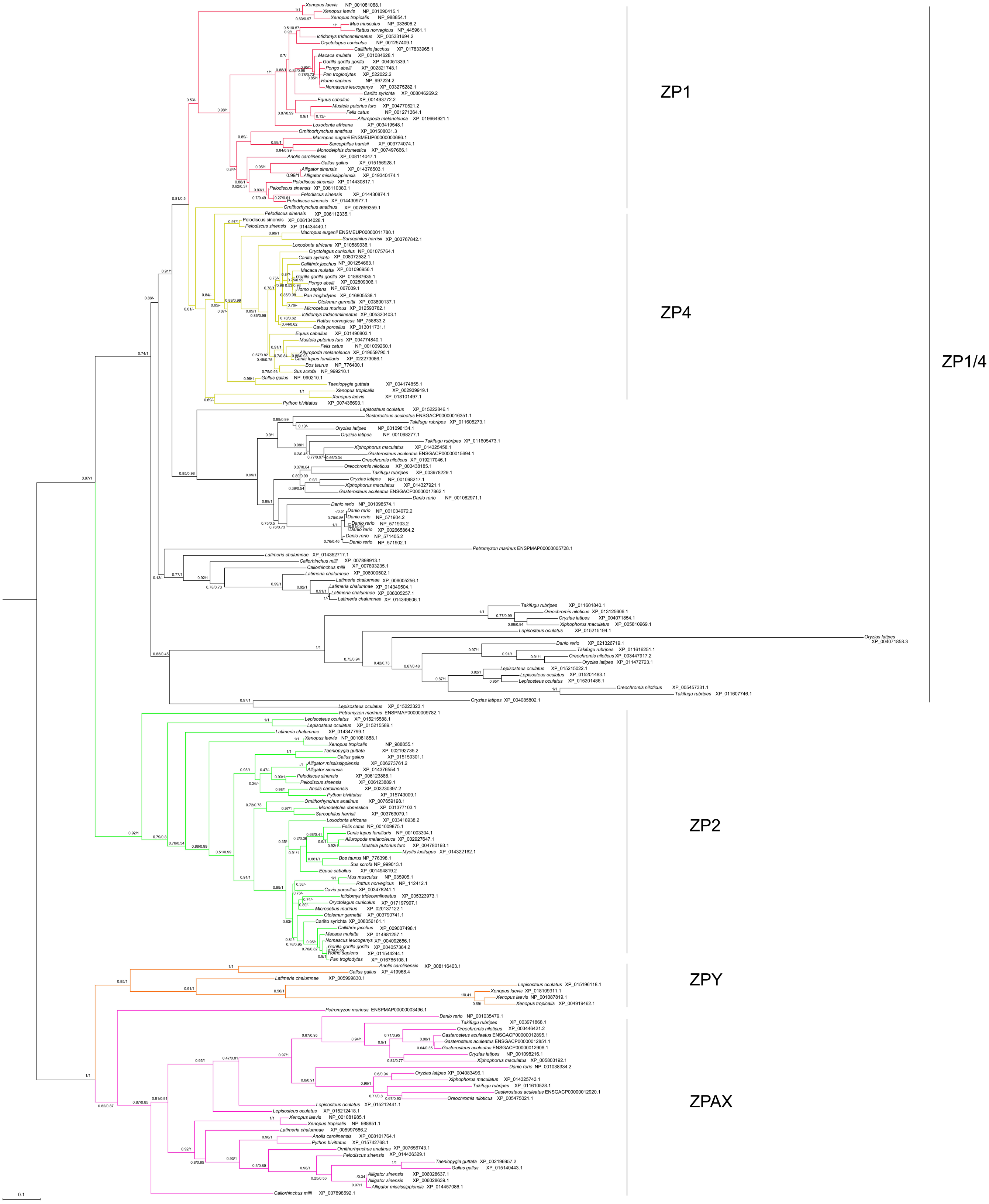

Fig. S4. Phylogeny of all ZP genes in Cluster 1 (ZP1/4, ZP2, ZPAX, and ZPY subfamilies) after excluding the fastest-evolving sites. The phylogenetic tree was inferred based on an aligned amino acid dataset with 173 sites. The numbers shown in the nodes correspond to the support values obtained with FastTree and PhyML. The scale bar indicates the average number of amino acid substitutions per site.

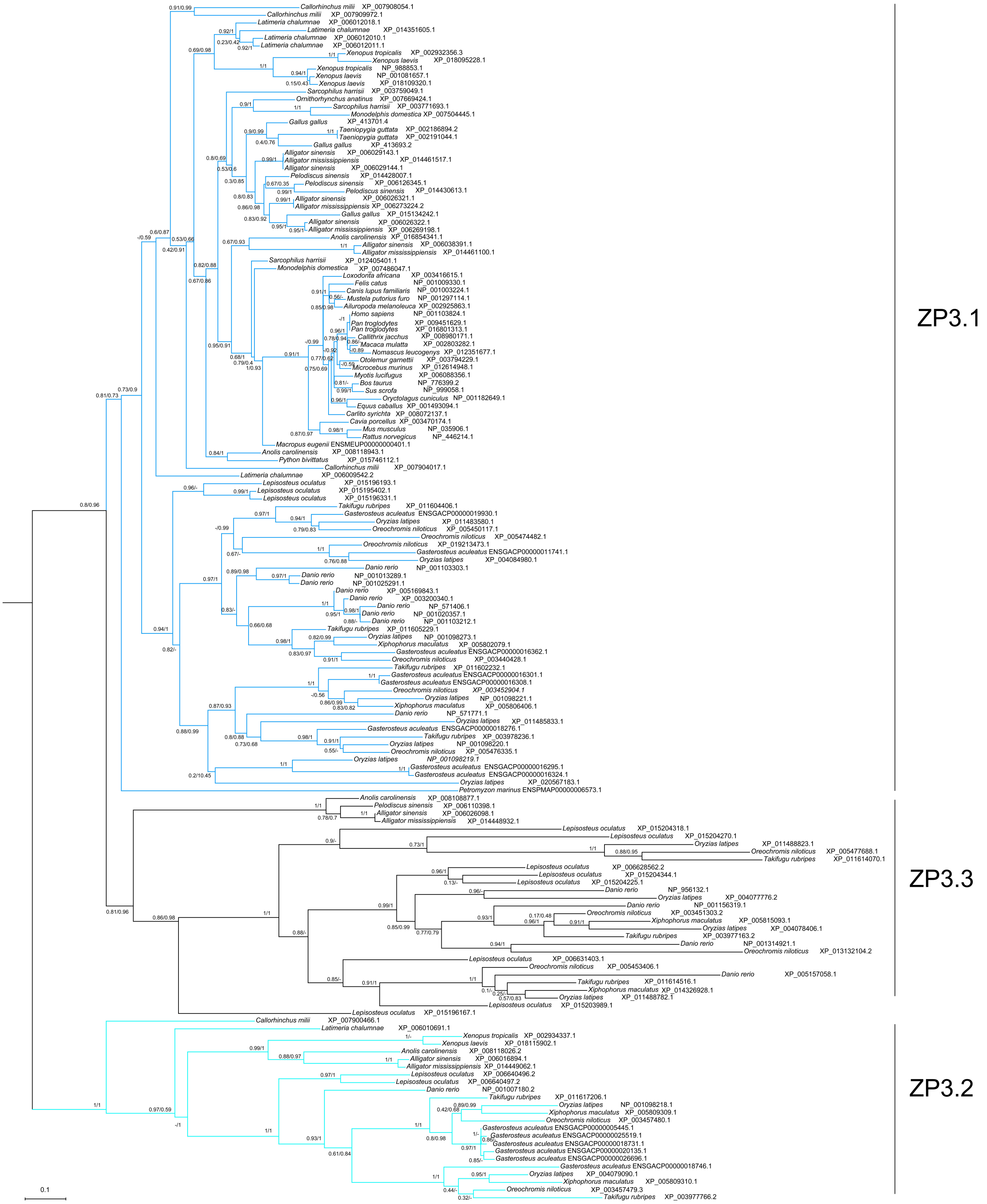

Fig. S5. Phylogeny of all ZP genes in Cluster 2 (ZP3.1, ZP3.2, and ZP3.3 subfamilies) after excluding the fastest-evolving sites. The phylogenetic tree was inferred based on an aligned amino acid dataset with 166 sites. The numbers shown in the nodes correspond to the support values obtained with FastTree and PhyML. The scale bar indicates the average number of amino acid substitutions per site.

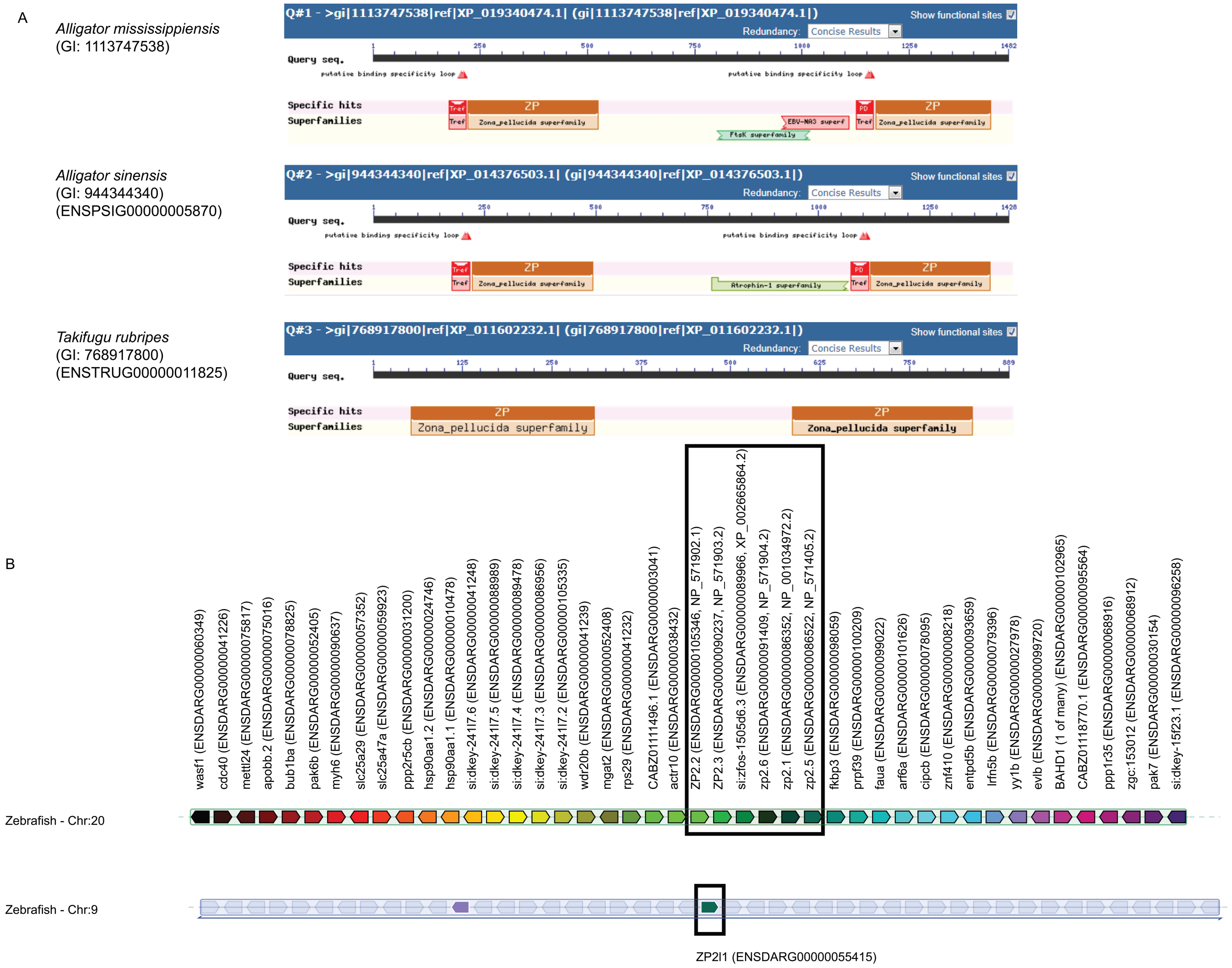

Fig. S6. (A) Two ZP modules characterized in *Alligator mississippiensis*, *Alligator sinensis*, and *Takifugu rubripes*.

(B) Tandem repeat of ZP1/4 in *Danio rerio*. Homologues of ZP1/4 in *Danio rerio* are indicated by the black box.

Biology Open • Supplementary information

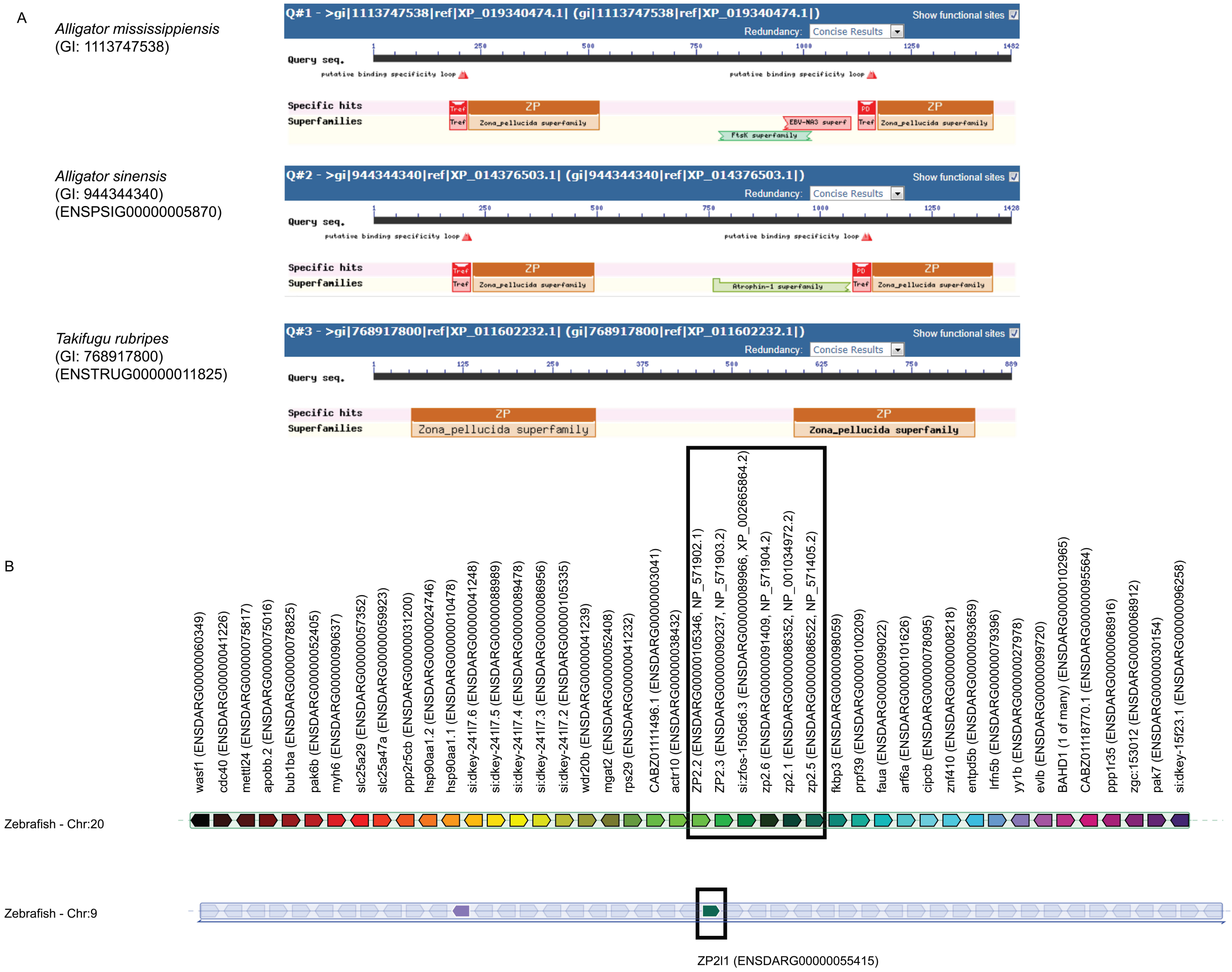

Fig. S7. Evidence of pseudogenes of the ZP3.2, ZP3.3 and ZPY genes. Stop codon(s) are marked in square boxes.

Biology Open • Supplementary information

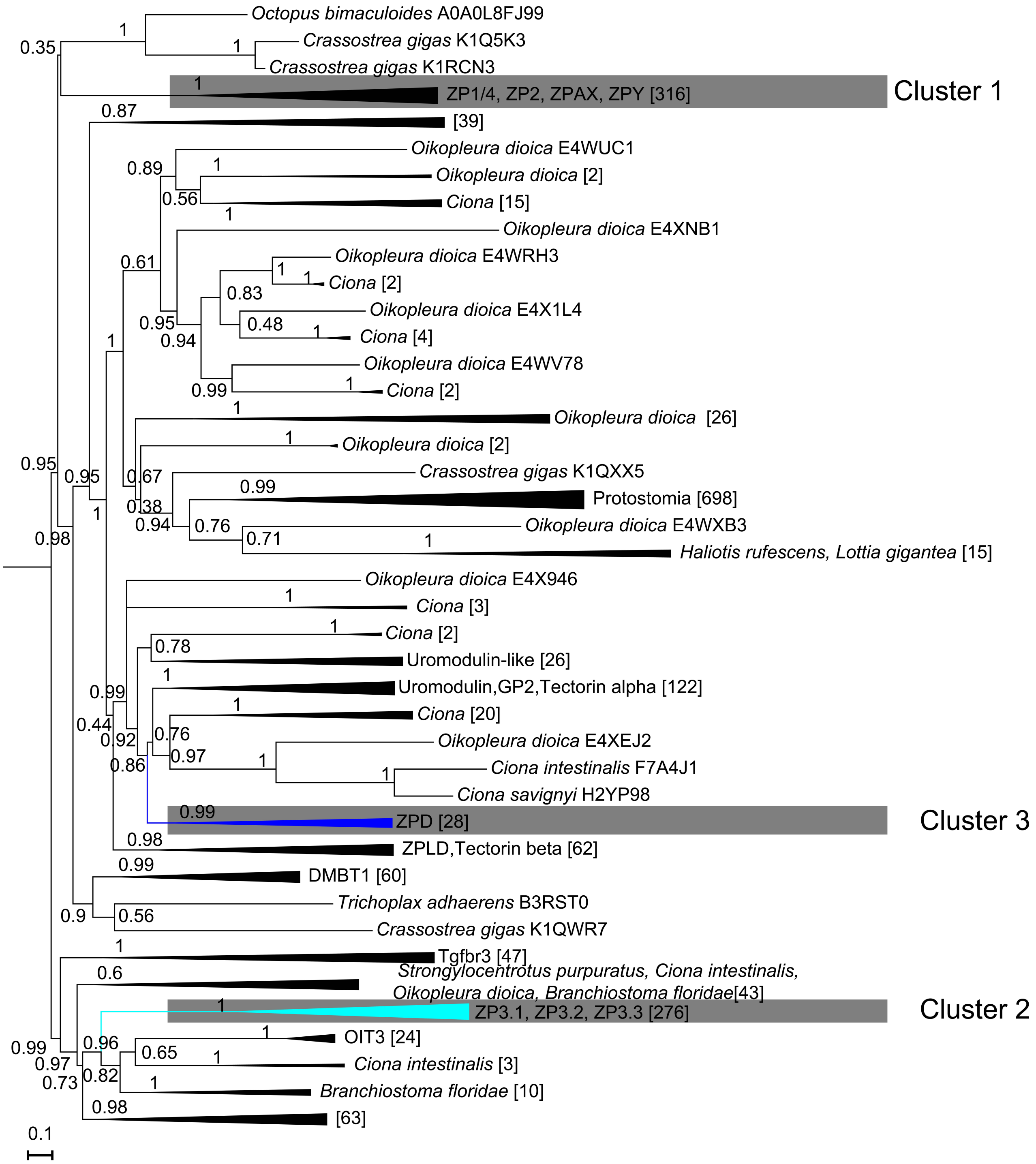

Fig. S8. Phylogeny of all ZP genes characterized in this study and representative ZP module-containing genes. The phylogenetic tree was inferred based on an aligned amino acid dataset with 160 sites. The numbers shown in the nodes correspond to the support values obtained with PhyML. The scale bars indicate the average number of amino acid substitutions per site.

Biology Open • Supplementary information

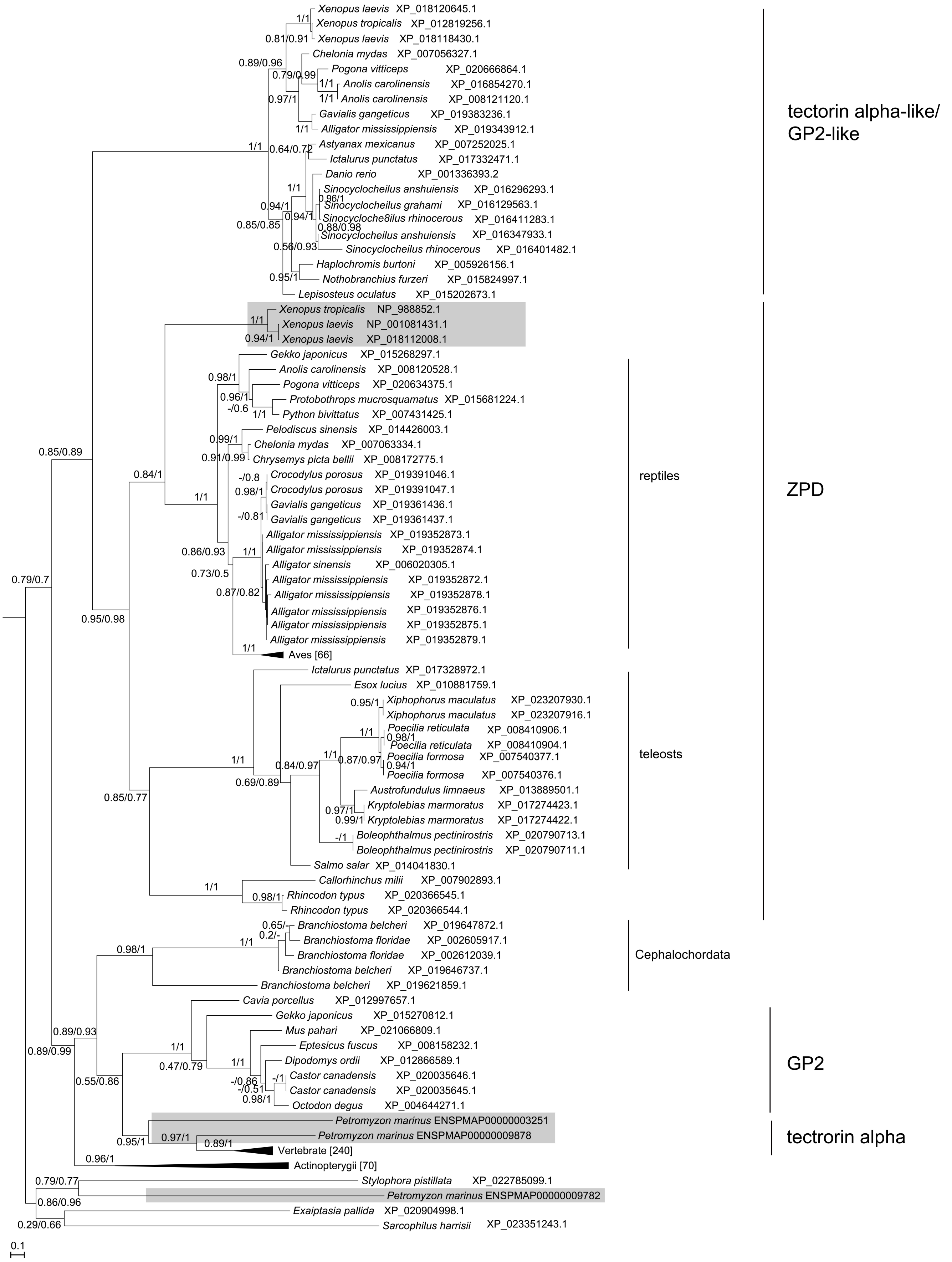

Biology Open • Supplementary information

Fig. S9. Phylogeny of ZPD genes and related ZP module-containing genes. The phylogenetic tree was inferred based on an aligned amino acid dataset with 287 sites and is shown rooted on the midpoint. The characterized ZPD genes of *Xenopus* and similar sequences of *Petromyzon marinus* are shown with a grey background. The numbers shown in the nodes correspond to support values of FastTree and PhyML. The scale bars indicate the average number of amino acid substitutions per site.
